# UV-B Perceived by the UVR8 Photoreceptor Inhibits Plant Thermomorphogenesis

**DOI:** 10.1016/j.cub.2016.11.004

**Published:** 2017-01-09

**Authors:** Scott Hayes, Ashutosh Sharma, Donald P. Fraser, Martine Trevisan, C. Kester Cragg-Barber, Eleni Tavridou, Christian Fankhauser, Gareth I. Jenkins, Keara A. Franklin

**Affiliations:** 1 School of Biological Sciences, Life Sciences Building, University of Bristol, Bristol BS8 1TQ, UK; 2 Plant Ecophysiology, Institute of Environmental Biology (IEB), Utrecht University, Padualaan 8, 3584 Utrecht, the Netherlands; 3 Centre for Integrative Genomics, Faculty of Biology and Medicine, University of Lausanne, 1015 Lausanne, Switzerland; 4 Department of Botany and Plant Biology, University of Geneva, Sciences III, 30 Quai E. Ansermet, 1211 Geneva 4, Switzerland; 5 Institute of Molecular, Cell and Systems Biology, College of Medical, Veterinary and Life Sciences, University of Glasgow, Glasgow G12 8QQ, UK

**Keywords:** UV-B, *Arabidopsis*, UVR8, high temperature, auxin, hypocotyl, HFR1, PIF4

## Abstract

Small increases in ambient temperature can elicit striking effects on plant architecture, collectively termed thermomorphogenesis [1]. In *Arabidopsis thaliana*, these include marked stem elongation and leaf elevation, responses that have been predicted to enhance leaf cooling [2, 3, 4, 5]. Thermomorphogenesis requires increased auxin biosynthesis, mediated by the bHLH transcription factor PHYTOCHROME-INTERACTING FACTOR 4 (PIF4) [6, 7, 8], and enhanced stability of the auxin co-receptor TIR1, involving HEAT SHOCK PROTEIN 90 (HSP90) [9]. High-temperature-mediated hypocotyl elongation additionally involves localized changes in auxin metabolism, mediated by the indole-3-acetic acid (IAA)-amido synthetase Gretchen Hagen 3 (GH3).17 [10]. Here we show that ultraviolet-B light (UV-B) perceived by the photoreceptor UV RESISTANCE LOCUS 8 (UVR8) [11] strongly attenuates thermomorphogenesis via multiple mechanisms inhibiting PIF4 activity. Suppression of thermomorphogenesis involves UVR8 and CONSTITUTIVELY PHOTOMORPHOGENIC 1 (COP1)-mediated repression of *PIF4* transcript accumulation, reducing PIF4 abundance. UV-B also stabilizes the bHLH protein LONG HYPOCOTYL IN FAR RED (HFR1), which can bind to and inhibit PIF4 function. Collectively, our results demonstrate complex crosstalk between UV-B and high-temperature signaling. As plants grown in sunlight would most likely experience concomitant elevations in UV-B and ambient temperature, elucidating how these pathways are integrated is of key importance to the understanding of plant development in natural environments.

## Highlights

• UVR8 activity inhibits auxin signaling and stem elongation at high temperature
• UVR8 acting with COP1 suppresses transcript abundance of the bHLH factor PIF4
• UV-B-mediated degradation of PIF4 is temperature dependent
• UV-B stabilizes the bHLH factor HFR1, which can bind to and inhibit PIF4 function

## Teaser

Hayes et al. show that low-dose UV-B, perceived by the UVR8 photoreceptor, is a potent inhibitor of high-temperature-induced stem elongation. This provides plants with an important braking mechanism in bright sunlight, preventing excessive elongation growth that could lead to stem lodging and critical reductions in root and leaf biomass.

## Results and Discussion

Growth in stressful environments, such as high temperature and vegetational shade, can trigger plant acclimation/escape responses involving rapid stem elongation at the expense of biomass production [12, 13]. A number of studies have identified molecular crosstalk between high temperature and light signaling via the red/far-red light-absorbing phytochrome photoreceptors [13]. More recently, cryptochrome 1 has been shown to physically interact with phytochrome-interacting factor 4 (PIF4) [14, 15] to regulate high-temperature-mediated hypocotyl elongation in blue light [14]. Although daily peaks in ultraviolet-B light (UV-B) levels correlate with temperature maxima in natural photoperiods [16], the integration of UV-B and thermomorphogenesis signaling pathways has remained largely unexplored. Following UV-B absorption, UV resistance locus 8 (UVR8) monomerizes and binds to the E3 ubiquitin ligase constitutively photomorphogenic 1 (COP1) to initiate downstream signaling [11].

**Figure 1:**
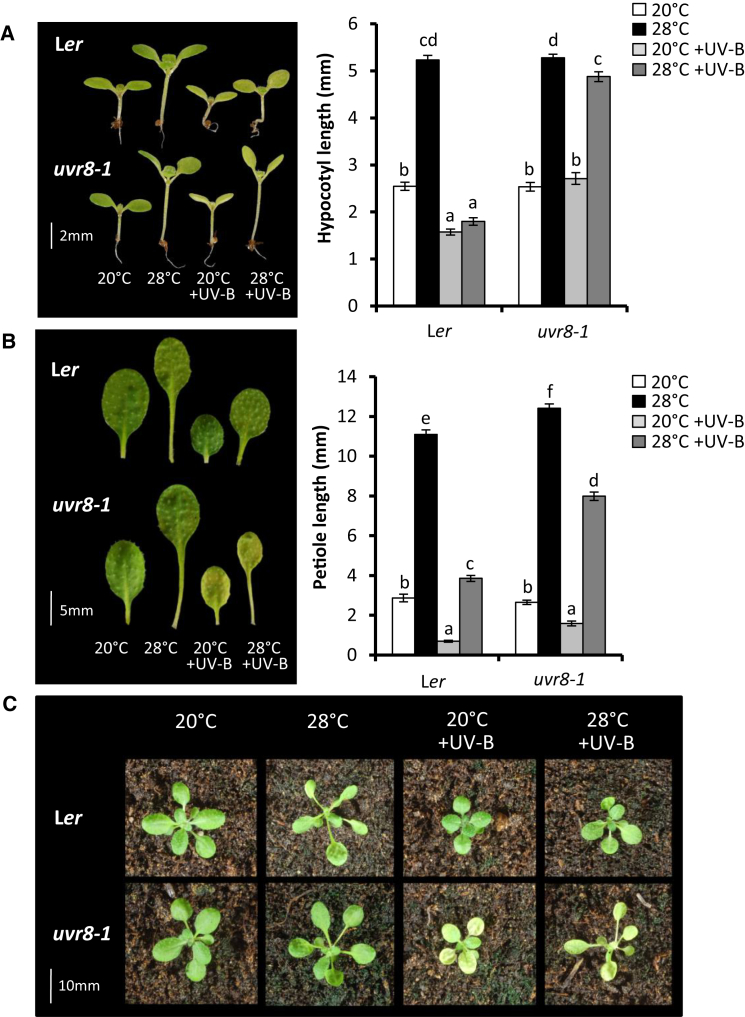
UV-B Perceived by UVR8 Inhibits High-Temperature-Induced Architectural Adaptations in *Arabidopsis* (A) Hypocotyl lengths of L*er* and *uvr8*-*1* seedlings grown in continuous light for 3 days at 20°C, before transfer to 20°C, 28°C, 20°C + UV-B, or 28°C + UV-B for a further 4 days. Data represent mean length (n = 40) ± SE. (B) Petiole length of leaf 4 of L*er* and *uvr8*-*1* plants grown for 10 days in 16 hr light/8 hr dark cycles at 20°C before transfer to 20°C, 28°C, 20°C + UV-B, or 28°C + UV-B for a further 9 days. Data represent mean length (n ≥ 23) ± SE. Different letters indicate statistically significant means (p < 0.05). Two-way ANOVA confirmed that there was a significant interaction between genotype and condition on petiole length (p < 0.001). (C) Representative rosettes of plants grown as in (B). See also Figure S1.

Here we show that low-dose UV-B provides a strong brake on high-temperature-induced hypocotyl elongation in seedlings (Figure 1A) and petiole elongation in adult plants (Figures 1B and 1C). UV-B-mediated inhibition of hypocotyl elongation at high temperature was observed in continuous light, 16 hr photoperiods, and 8 hr photoperiods, suggesting no photoperiodic specificity to the response (Figures 1A, S1A, and S1B). Considerable high-temperature-mediated stem elongation responses were observed in the *uvr8-1* mutant in the presence of UV-B, demonstrating that the inhibition effects observed are predominantly photomorphogenic responses mediated by UVR8 (Figures 1A–1C). Some UVR8-independent, UV-B-mediated inhibitions of hypocotyl and petiole elongation were, however, recorded. In addition to changes in petiole length, a UVR8-mediated suppression of high-temperature-induced leaf hyponasty was observed in UV-B (Figure S1C). UV-B treatment decreased leaf area independently of UVR8 at 20°C and 28°C. A smaller decrease was observed following high-temperature treatment in wild-type (WT) plants, but not in *uvr8* mutants. When UV-B and temperature were applied simultaneously, elevated temperature rescued the small leaf phenotype induced by UV-B in a UVR8-dependent manner (Figure S1D). UV-B-induced reductions in leaf area are complex and likely to involve stress signaling pathways in addition to UVR8 signaling [17]. Leaf area phenotypes may therefore reflect enhanced repair of UV-B-induced DNA damage at high temperature [18, 19].

**Figure 2:**
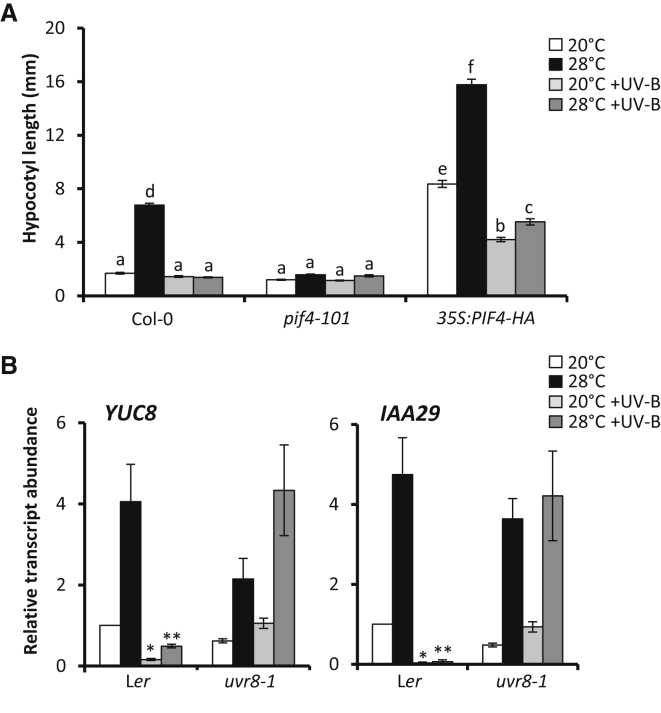
UV-B Perceived by UVR8 Inhibits PIF4 Activity and Auxin Signaling at High Temperature (A) Hypocotyl lengths of Col-0, *pif4*-*101*, and *35S:PIF4*-*HA* seedlings grown in continuous light for 3 days at 20°C, before transfer to 20°C, 28°C, 20°C + UV-B, or 28°C + UV-B for a further 4 days (n ≤ 27; ±SE). Different letters indicate statistically significant means (p < 0.05). (B) Relative transcript abundance of *YUC8* and *IAA29* in L*er* and *uvr8*-*1* seedlings grown for 10 days in 16 hr light/8 hr dark cycles at 20°C, before transfer at dawn to the indicated conditions for 4 hr (n = 3; ±SE; ^∗^significant UV-B-mediated decrease in transcript abundance when compared to 20°C, p < 0.05; ^∗∗^significant UV-B-mediated decrease in transcript abundance when compared to 28°C, p < 0.05). See also Figure S1.

Transfer of plants to high temperature transiently increases *PIF4* transcript abundance [6, 7, 8, 20] and promotes the accumulation of phosphorylated PIF4 protein [12]. In diurnal cycles, warm temperatures inhibit the transcriptional regulator EARLY FLOWERING 3 (ELF3), relieving *PIF4* repression at night [21, 22, 23]. PIF4 promotes the expression of auxin biosynthesis genes [8, 24], including *YUCCA8* (*YUC8*), which encodes a key rate-limiting enzyme in tryptophan-dependent auxin biosynthesis [25, 26]. High temperature therefore elevates free indole-3-acetic acid (IAA, the major natural auxin) levels and the expression of auxin-responsive genes, such as *IAA29* [2, 6, 8, 24]. As expected, no significant high-temperature-induced hypocotyl elongation was evident in *pif4* mutants in our conditions (Figure 2A) [6, 7, 8]. UV-B strongly suppressed the elongated phenotype of *PIF4* overexpressor seedlings at 20°C and 28°C, suggesting that UV-B may inhibit PIF4 activity (Figure 2A). In support of this idea, UV-B inhibited the accumulation of *YUC8* and *IAA29* transcript abundance at both temperatures (Figure 2B). Consistent with hypocotyl elongation data (Figure 1A), UV-B-mediated suppression of auxin biosynthesis/signaling genes was dependent upon the presence of UVR8, confirming the response to be photomorphogenic (Figure 2B). No high-temperature-mediated increase in *IAA29* transcript was observed in *pif4* mutants. PIF4 overexpressor seedlings displayed elevated levels of *IAA29* transcript, which were supressed by UV-B (Figure S1E).

**Figure 3:**
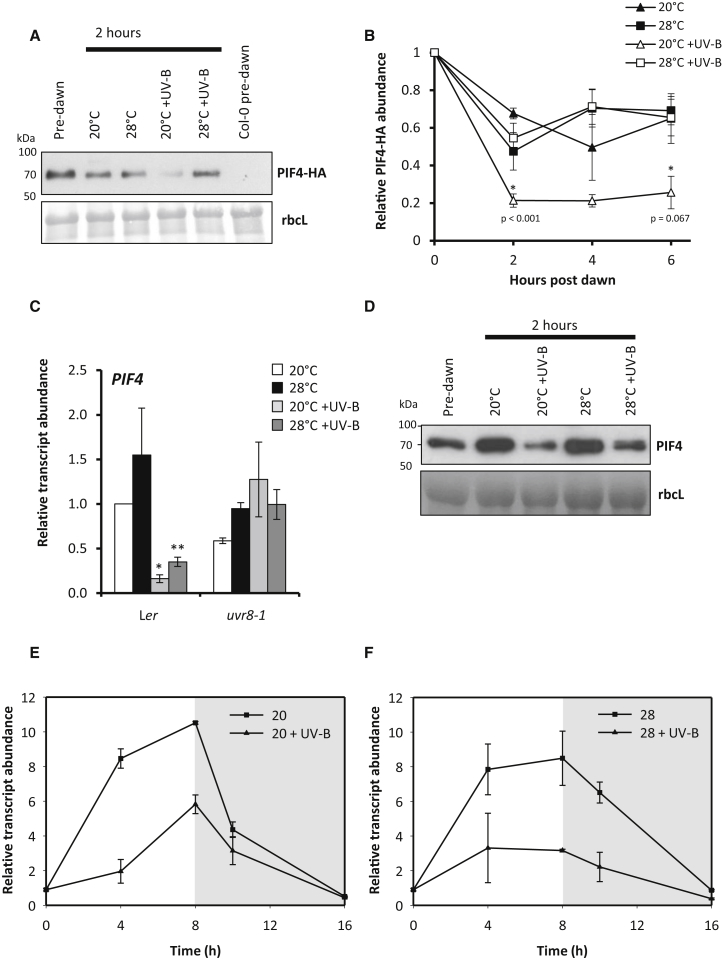
UV-B Inhibits *PIF4* Transcript Accumulation in a UVR8-Dependent Manner and Promotes PIF4 Degradation in a Temperature-Conditional Manner (A) PIF4-HA abundance in *35S*:*PIF4*-*HA* seedlings grown for 10 days in 16 hr light/8 hr dark cycles at 20°C, harvested before dawn and 2 hr after dawn following transfer to the stated conditions. Col-0 serves as a negative control. Ponceau stain of Rubisco large subunit (rbcL) serves as a loading control. (B) Time course of plants grown and treated as in (A). Relative protein abundance was normalized to Ponceau staining of the Rubisco large subunit, then expressed as a value relative to pre-dawn levels (n = 3; ±SE). Asterisks denote a significant difference between UV-B- and white light (WL)-treated controls at their respective temperatures. (C) *PIF4* transcript abundance in L*er* and *uvr8-1* seedlings grown as in (A) and harvested at 4 hr (^∗^significant UV-B-mediated decrease in transcript abundance when compared to 20°C, p < 0.05; ^∗∗^significant UV-B-mediated decrease in transcript abundance when compared to 28°C, p < 0.05). (D) Representative blot showing PIF4 abundance in L*er* grown as in (A) at the 2 hr time point using anti-PIF4 antibody. Ponceau stain of Rubisco large subunit (rbcL) serves as a loading control. (E and F) Time course of *PIF4* transcript abundance. Seedlings were grown for 10 days in 8 hr light/16 hr dark cycles at 20°C. On day 11, plants were transferred to either (E) 20°C or (F) 28°C ± UV-B. UV-B treatment was maintained for the duration of the photoperiod and plants harvested at the times shown. All values are normalized to time 0. The mean of two biological repeats are shown ± SD. See also Figures S2 and S3.

UV-B has previously been shown to inhibit auxin biosynthesis in simulated canopy shade (low red-to-far red ratio light; low R:FR), by promoting PIF4/PIF5 degradation and stabilizing DELLA proteins [27]. The latter inhibit PIF function through heterodimerization [28, 29]. We therefore analyzed the stability of constitutively expressed, hemagglutinin (HA)-tagged PIF4 in our conditions. In agreement with previous observations at 20°C, UV-B treatment resulted in rapid PIF4-HA degradation (Figures 3A and 3B) [27]. Intriguingly, no UV-B-mediated degradation of PIF4-HA was observed at 28°C, suggesting a temperature-dependent component to this response (Figures 3A and 3B). We next investigated UV-B-mediated suppression of thermomorphogenesis in a DELLA quintuple mutant, deficient in all DELLA proteins [6]. Despite showing longer hypocotyls than WT plants in all experimental conditions, strong UV-B-mediated inhibition of hypocotyl elongation was observed in *della*-null mutants at high temperature, confirming that UV-B-mediated stabilization of DELLAs does not constitute an underlying regulatory mechanism in the inhibition of thermomorphogenesis (Figure S2A). Together, these data suggest that the dominance of regulatory components controlling UV-B-mediated hypocotyl inhibition differs between thermomorphogenesis and shade avoidance.

We next investigated the effect of UV-B on *PIF4* transcript abundance. UV-B strongly inhibited *PIF4* transcript accumulation at 20°C and 28°C in a UVR8-dependent manner (Figure 3C). Mutants deficient in the UVR8-binding protein COP1 showed significantly reduced *PIF4* transcript in the absence of UV-B and insensitivity to UV-B treatment at both temperatures (Figure S2B). Such data suggest a fundamental requirement for COP1 in promoting *PIF4* transcript accumulation. Consistent with this observation and previous studies [20], we observed no thermomorphogenesis in *cop1* mutants (Figure S2C). Plants expressing a constitutively dimeric form of UVR8 in the *uvr8*-*1* background (*uvr8*-*1*/GFP-UVR8^W285F^), which is unable to bind COP1 [30], displayed no UV-B-mediated inhibition of thermomorphogenesis (Figure S2D). This supports the hypothesis that UVR8 monomerization and UVR8-COP1 binding is required for this response. The effect of reduced *PIF4* transcript levels on PIF4 protein abundance was investigated by western blotting of native PIF4, using a polyclonal PIF4 antibody. This antibody recognized PIF4 when tested on a range of mutant and transgenic lines (Figure S2E). UV-B treatment strongly decreased PIF4 abundance at both temperatures, suggesting that UVR8-mediated suppression of *PIF4* transcript abundance reduces PIF4 protein (Figure 3D).

The transcriptional regulation of *PIF4* has been shown to involve the regulatory proteins ELF3 and ELONGATED HYPOCOTYL 5 (HY5) [20, 21]. In day/night cycles, ELF3 supresses the transcription of *PIF4* in the early evening, promoting PIF4 accumulation and hypocotyl elongation toward the end of the night [23]. As high temperatures suppress ELF3 binding to the *PIF4* promoter [21], it has been proposed that (in short days at least) reduced ELF3-mediated repression of *PIF4* transcript accumulation drives high-temperature-mediated architectural changes. We therefore investigated the consequence of daytime UV-B supplementation on nighttime *PIF4* transcript levels at 20°C and 28°C. Plants were grown in short (8 hr) photoperiods, and *PIF4* levels were quantified throughout the day and early night. In the absence of UV-B, *PIF4* transcript showed the expected early night suppression at 20°C (Figure 3E) [21, 23, 31]. UV-B strongly reduced *PIF4* transcript abundance during the day, maintaining low *PIF4* levels throughout the early night, before eventually reaching similar levels to untreated plants by 8 hr of dark. At 28°C, *PIF4* transcript levels were higher than at 20°C during the early night, consistent with reduced ELF3 function [21]. UV-B treatment still, however, resulted in a sustained suppression of *PIF4* transcript levels across the entire time course (Figure 3F). Despite showing a higher abundance of *PIF4* transcript than WT plants at dawn [21], *elf3-1* mutants displayed UV-B-mediated *PIF4* suppression at both temperatures (Figures S3A and S3B). These data suggest that the inhibition of *PIF4* transcript abundance by UV-B can occur independently of ELF3.

**Figure 4:**
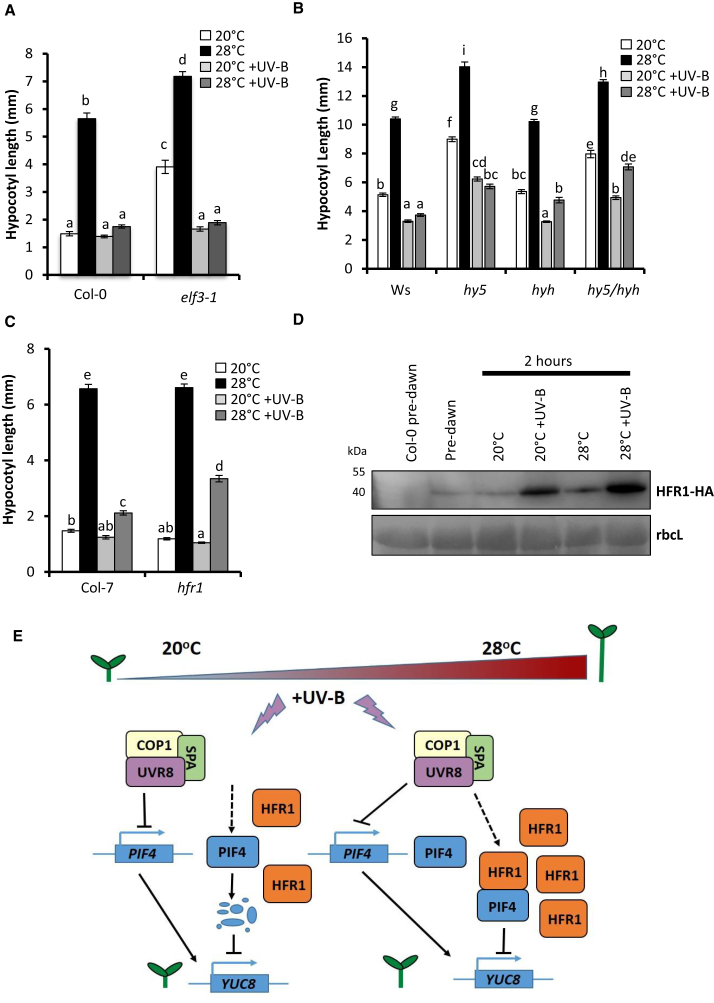
UV-B-Mediated Stabilization of HFR1 Suppresses PIF4 Activity at 28°C (A–C) Hypocotyl lengths of (A) Col-0 and *elf3*-*1*; (B) Ws, *hy5*, *hyh*, and *hy5*/*hyh*; and (C) Col-7 and *hfr1* seedlings grown in continuous light for 3 days at 20°C, before transfer to 20°C, 28°C, 20°C + UV-B, or 28°C + UV-B for a further 4 days. Data represent mean values (n = 40) ± SE. Different letters indicate statistically significant means (p < 0.05). Two-way ANOVA confirmed an interaction between genotype^∗^condition on hypocotyl length between Col-7 and *hfr1* plants (p < 0.001). (D) Representative blot showing HFR1-HA abundance in pH*FR1*:*HFR1*-*HA* seedlings grown for 10 days in 16 hr light/8 hr dark cycles at 20°C, following 2 hr transfer to the stated conditions using an anti-HA antibody. (E) Hypothetical model depicting UV-B-mediated inhibition of hypocotyl elongation at different temperatures. At 20°C, UV-B perceived by UVR8 inhibits *PIF4* transcript accumulation in a response requiring COP1. This reduces PIF4 protein abundance. Simultaneously, UV-B drives degradation of PIF4 protein and stabilizes HFR1. At 28°C, UV-B perceived by UVR8 inhibits *PIF4* transcript abundance, in a response requiring COP1. This reduces PIF4 protein accumulation. PIF4 is protected from UV-B-induced degradation at elevated temperature, but its transcriptional activity is inhibited by high HFR1 levels. The abundance of HFR1 increases at 28°C. In UV-B, UVR8 sequesters COP1, inhibiting COP1-mediated HFR1 degradation. A role for HYH in the UV-B-mediated inhibition of hypocotyl elongation was additionally observed at high temperature, although no known mechanism exists for HYH regulation of PIF4 activity. Collectively, UV-B inhibits hypocotyl elongation by reducing PIF4 abundance and activity, thereby limiting auxin biosynthesis. The relative contributions of different regulatory mechanisms to this overall response are dependent on ambient temperature. See also Figures S2–S4.

It is likely that UV-B also regulates PIF4 activity at the post-translational level (as evidenced by strong inhibition of hypocotyl elongation in *PIF4* overexpressor seedlings in Figure 2A). ELF3 and HY5 have been shown to inhibit PIF4 activity by direct physical interaction [31] and antagonistic promoter binding [32], respectively. To investigate post-translational regulation by ELF3, we investigated the effect of UV-B on hypocotyl inhibition in *elf3-1* mutants at 20°C and 28°C. Consistent with previous reports, *elf3-1* mutants displayed elongated hypocotyls at 20°C and an exaggerated elongation response to high temperature (Figures 4A and S3C) [21, 31]. These phenotypes were abolished in the presence of UV-B, consistent with a role for UV-B signaling in inhibiting PIF4 function. As UV-B fully inhibited thermomorphogenesis in *elf3* mutants (Figures 4A and S3C), it is unlikely that ELF3 forms a regulatory component of this response. We therefore investigated the role of HY5 and its close relative, HY5 HOMOLOG (HYH). The expression of both is strongly increased in UV-B [33, 34, 35]. Single and double *hy5/hyh* mutants displayed significant UV-B-mediated hypocotyl inhibition at 20°C and 28°C (Figure 4B). High-temperature-mediated hypocotyl elongation was completely inhibited by UV-B in WT and *hy5* mutants, suggesting that HY5 is not required for the inhibition response. Conversely, some high-temperature-mediated hypocotyl elongation was observed in *hyh* and *hy5/hyh* mutants in the presence of UV-B, suggesting that HYH contributes to UV-B-mediated thermomorphogenesis inhibition (Figure 4B). No role for HY5 or HYH could be identified in the UV-B-mediated suppression of *PIF4* transcript accumulation (Figure S3D).

The RNA-binding protein FCA has been shown to attenuate hypocotyl elongation at high temperature by promoting the dissociation of PIF4 from *YUC8* chromatin [36]. High temperature increases *FCA* transcript levels 2-fold but does not affect protein stability [36]. A similar high-temperature effect on *FCA* transcript levels was observed in our experimental conditions. This was inhibited by UV-B in a UVR8-dependent manner, suggesting that FCA does not form a component of UV-B-mediated hypocotyl inhibition at high temperature (Figure S4A). In low R:FR, PIF function is antagonized in a negative feedback loop by the HLH proteins PHYTOCHROME RAPIDLY REGULATED 1 (PAR1) and PAR2 and the bHLH protein LONG HYPOCOTYL IN FAR RED (HFR1), which form competitive heterodimers unable to bind DNA [37, 38, 39]. Inhibition of PIF4 activity by HFR1 has also been reported in monochromatic FR and blue light [12, 40]. Mutants deficient in PAR2 (*par2-1*) displayed exaggerated hypocotyl elongation at high temperature, suggesting a role for PAR2 in the suppression of this response in white light (Figure S4B). Despite this, both *par2-1* and transgenic plants containing reduced transcripts of both *PAR1* and *PAR2* (*PAR1 RNAi*) [39] displayed full UV-B-mediated inhibition of thermomorphogenesis, suggesting neither to be essential for this response (Figure S4B). Indeed, UV-B perceived by UVR8 strongly inhibited *PAR1* and *PAR2* transcript accumulation (Figures S4C and S4D), consistent with their roles as PIF4 target genes.

A partial inhibition of thermomorphogenesis was, however, observed in *hfr1* mutants in the presence of UV-B, suggesting a regulatory role for this protein (Figure 4C). In agreement with previous observations, *HFR1* transcript abundance increased significantly at high temperature (Figure S4E) [12] but was strongly suppressed by UV-B in a UVR8-dependent manner (Figure S4E), consistent with the role of *HFR1* as a PIF4 target gene [38]. We therefore investigated the effect of UV-B on HFR1 protein stability. HFR1 levels increased following transfer to high temperature (Figure 4D) [12] and were strongly stabilized by UV-B (Figure 4D). It is possible that HFR1 binding protects PIF4 from UV-B-induced degradation. Collectively, our data suggest that UV-B-mediated stabilization of HFR1 contributes to the suppression of PIF4 activity and inhibition of hypocotyl elongation in these conditions.

### Conclusions

Here we demonstrate that UV-B is a potent inhibitor of plant thermomorphogenesis (summarized in Figure 4E). Low-dose UV-B supplementation promoted the degradation of PIF4 protein at 20°C, but not 28°C. At high temperature, UV-B, perceived by UVR8, strongly inhibited *PIF4* transcript accumulation, resulting in low PIF4 levels and reduced expression of auxin biosynthesis/signaling genes. No role for the characterized PIF4 transcriptional regulators ELF3 or HY5 [20, 21] could be identified in this response. In the absence of COP1, *PIF4* transcript levels remained low and insensitive to UV-B, suggesting a role for this protein in regulating *PIF4* transcript abundance. Reduced *PIF4* transcript has also been reported in *de-etiolated 1* (*det-1*) mutants, deficient in an enhancer of COP1 activity [20]. Mutants deficient in COP1 do not elongate at high temperature [20] (Figure S2B), so the role of COP1 in the UV-B-mediated inhibition of this response could not be directly tested. GFP-UVR8^W285F^ plants express a constitutively dimerized UVR8 in the *uvr8-1* background, which is unable to bind COP1 and initiate photomorphogenic signaling [30]. High-temperature-mediated hypocotyl elongation was similar in UV-B-treated GFP-UVR8^W285F^ and *uvr8* mutants, suggesting that UVR8 inhibits thermomorphogenesis via its established photoreceptor activity.

UV-B-mediated suppression of hypocotyl elongation in *PIF4* overexpressor plants suggested that UVR8 inhibits PIF4 activity in addition to repressing transcript abundance. Two recent studies have shown that CRY1 and CRY2 physically interact with PIF4 in blue light to inhibit thermomorphogenesis and shade avoidance [14, 15]. By contrast, UVR8 appears not to physically interact with PIFs [27]. UVR8 does, however, directly interact with COP1 [11]. It is possible that in the presence of UV-B, UVR8 sequesters COP1, reducing its E3 ubiquitin ligase activity and enabling the accumulation of PIF4-negative regulators. Mutant analyses showed that the majority of known PIF4 inhibitors (DELLAs, ELF3, HY5, and PARs) does not have a dominant role in UVR8-mediated inhibition of thermomorphogenesis [28, 29, 31, 32, 39], although we cannot rule out functional redundancy between these regulators. Intriguingly, some role was identified for HYH, highlighting different regulatory capabilities between HY5 and HYH in these conditions. The mechanism by which HYH inhibits PIF4 function is unclear, but it may compete for *PIF4* target promoters at higher temperatures. A clear role was, however, identified for HFR1, which is known to antagonize high-temperature-mediated elongation growth in blue light [12]. High temperature and UV-B both stabilized HFR1 protein, which can inhibit PIF4 activity through heterodimer formation [38]. It is therefore likely that high HFR1 levels contribute to the UV-B-mediated suppression of thermomorphogenesis.

Collectively, our data support the existence of an overarching mechanism through which UV-B inhibits hypocotyl elongation in *Arabidopsis*. This involves the repression of PIF abundance and activity, which subsequently prevents the upregulation of auxin biosynthesis. The relative contributions of different regulatory components do, however, appear to change with environmental context [27]. Here we show that the molecular mechanisms controlling UV-B-mediated suppression of hypocotyl elongation vary with growth temperature. UVR8/COP1-mediated suppression of *PIF4* transcript accumulation appears to strongly inhibit PIF4 protein accumulation at 20°C and 28°C (Figures 3C–3F and S2B). PIF4 protein is additionally degraded by UV-B treatment at cooler temperatures (Figures 3A and 3B) [27]. At 28°C, PIF4 protein is protected from UV-B-induced degradation (Figures 3A and 3B) but has severely reduced function. We hypothesize that this results, at least in part, from high HFR1 levels in these conditions (Figure 4D). This assumption is supported by the reduced UV-B-mediated hypocotyl growth inhibition observed in *hfr1* mutants at 28°C (Figure 4C).

Elongation growth at high temperature may facilitate plant fitness through enhancing leaf cooling capacity [2, 3, 4, 5]. Excessive stem growth at the expense of leaf and root development could, however, prove detrimental to plant survival, by critically reducing biomass and increasing lodging susceptibility. Our data suggest that enhanced UV-B absorption by leaves in direct sunlight would antagonize the effect of warming, thereby constraining stem elongation. It is therefore of particular relevance that daily peaks in UV-B and temperature coincide [16]. Caution must therefore be applied when interpreting thermomorphogenesis studies conducted in laboratory growth cabinets and glasshouses, which often contain little or no UV-B. Analysis of PIF function in natural canopies with fluctuating light and temperature conditions will be a key area of future research.

## Author Contributions

S.H., A.S., and D.P.F. designed and performed experiments and analyzed data. C.K.C.-B. performed experiments. M.T. and E.T. provided new material and performed experiments. C.F. designed experiments and interpreted data. K.A.F. and G.I.J. supervised the project and wrote the manuscript together with S.H., A.S., and D.P.F.

## Supplemental Information

Document S1. Supplemental Experimental Procedures and Figures S1–S4

Document S2. Article plus Supplemental Information

## References

[1] Quint M., Delker C., Franklin K.A., Wigge P.A., Halliday K.J., van Zanten M. (2016). Molecular and genetic control of plant thermomorphogenesis. Nat. Plants.

[2] Gray W.M., Östin A., Sandberg G., Romano C.P., Estelle M. (1998). High temperature promotes auxin-mediated hypocotyl elongation in Arabidopsis. Proc. Natl. Acad. Sci. USA.

[3] van Zanten M., Voesenek L.A.C.J., Peeters A.J.M., Millenaar F.F. (2009). Hormone- and light-mediated regulation of heat-induced differential petiole growth in Arabidopsis. Plant Physiol..

[4] Crawford A.J., McLachlan D.H., Hetherington A.M., Franklin K.A. (2012). High temperature exposure increases plant cooling capacity. Curr. Biol..

[5] Bridge L.J., Franklin K.A., Homer M.E. (2013). Impact of plant shoot architecture on leaf cooling: a coupled heat and mass transfer model. J. R. Soc. Interface.

[6] Koini M.A., Alvey L., Allen T., Tilley C.A., Harberd N.P., Whitelam G.C., Franklin K.A. (2009). High temperature-mediated adaptations in plant architecture require the bHLH transcription factor PIF4. Curr. Biol..

[7] Stavang J.A., Gallego-Bartolomé J., Gómez M.D., Yoshida S., Asami T., Olsen J.E., García-Martínez J.L., Alabadí D., Blázquez M.A. (2009). Hormonal regulation of temperature-induced growth in Arabidopsis. Plant J..

[8] Sun J., Qi L., Li Y., Chu J., Li C. (2012). PIF4-mediated activation of YUCCA8 expression integrates temperature into the auxin pathway in regulating arabidopsis hypocotyl growth. PLoS Genet..

[9] Wang R., Zhang Y., Kieffer M., Yu H., Kepinski S., Estelle M. (2016). HSP90 regulates temperature-dependent seedling growth in Arabidopsis by stabilizing the auxin co-receptor F-box protein TIR1. Nat. Commun..

[10] Zheng Z., Guo Y., Novák O., Chen W., Ljung K., Noel J.P., Chory J. (2016). Local auxin metabolism regulates environment-induced hypocotyl elongation. Nat. Plants.

[11] Rizzini L., Favory J.-J., Cloix C., Faggionato D., O’Hara A., Kaiserli E., Baumeister R., Schäfer E., Nagy F., Jenkins G.I., Ulm R. (2011). Perception of UV-B by the Arabidopsis UVR8 protein. Science.

[12] Foreman J., Johansson H., Hornitschek P., Josse E.M., Fankhauser C., Halliday K.J. (2011). Light receptor action is critical for maintaining plant biomass at warm ambient temperatures. Plant J..

[13] Franklin K.A., Toledo-Ortiz G., Pyott D.E., Halliday K.J. (2014). Interaction of light and temperature signalling. J. Exp. Bot..

[14] Ma D., Li X., Guo Y., Chu J., Fang S., Yan C., Noel J.P., Liu H. (2016). Cryptochrome 1 interacts with PIF4 to regulate high temperature-mediated hypocotyl elongation in response to blue light. Proc. Natl. Acad. Sci. USA.

[15] Pedmale U.V., Huang S.S., Zander M., Cole B.J., Hetzel J., Ljung K., Reis P.A.B., Sridevi P., Nito K., Nery J.R. (2016). Cryptochromes interact directly with PIFs to control plant growth in limiting blue light. Cell.

[16] Findlay K.M.W., Jenkins G.I. (2016). Regulation of UVR8 photoreceptor dimer/monomer photo-equilibrium in Arabidopsis plants grown under photoperiodic conditions. Plant Cell Environ..

[17] Robson T.M., Klem K., Urban O., Jansen M.A.K. (2015). Re-interpreting plant morphological responses to UV-B radiation. Plant Cell Environ..

[18] Li S.S., Wang Y., Björn L.O. (2004). Effects of temperature on UV-B-induced DNA damage and photorepair in *Arabidopsis thaliana*. J. Environ. Sci. (China).

[19] Takeuchi Y., Ikeda S., Kasahara H. (1993). Dependence on wavelength and temperature of growth inhibition induced by UV-B irradiation. Plant Cell Physiol..

[20] Delker C., Sonntag L., James G.V., Janitza P., Ibañez C., Ziermann H., Peterson T., Denk K., Mull S., Ziegler J. (2014). The DET1-COP1-HY5 pathway constitutes a multipurpose signaling module regulating plant photomorphogenesis and thermomorphogenesis. Cell Rep..

[21] Box M.S., Huang B.E., Domijan D., Jaeger K.E., Khattak A.K., Yoo S.J., Sedivy E.L., Jones D.M., Hearn T.J., Webb A.A. (2015). *ELF3* controls thermoresponsive growth in Arabidopsis. Curr. Biol..

[22] Mizuno T., Nomoto Y., Oka H., Kitayama M., Takeuchi A., Tsubouchi M., Yamashino T. (2014). Ambient temperature signal feeds into the circadian clock transcriptional circuitry through the EC night-time repressor in Arabidopsis thaliana. Plant Cell Physiol..

[23] Nusinow D.A., Helfer A., Hamilton E.E., King J.J., Imaizumi T., Schultz T.F., Farré E.M., Kay S.A. (2011). The ELF4-ELF3-LUX complex links the circadian clock to diurnal control of hypocotyl growth. Nature.

[24] Franklin K.A., Lee S.H., Patel D., Kumar S.V., Spartz A.K., Gu C., Ye S., Yu P., Breen G., Cohen J.D. (2011). Phytochrome-interacting factor 4 (PIF4) regulates auxin biosynthesis at high temperature. Proc. Natl. Acad. Sci. USA.

[25] Mashiguchi K., Tanaka K., Sakai T., Sugawara S., Kawaide H., Natsume M., Hanada A., Yaeno T., Shirasu K., Yao H. (2011). The main auxin biosynthesis pathway in Arabidopsis. Proc. Natl. Acad. Sci. USA.

[26] Won C., Shen X., Mashiguchi K., Zheng Z., Dai X., Cheng Y., Kasahara H., Kamiya Y., Chory J., Zhao Y. (2011). Conversion of tryptophan to indole-3-acetic acid by TRYPTOPHAN AMINOTRANSFERASES OF ARABIDOPSIS and YUCCAs in Arabidopsis. Proc. Natl. Acad. Sci. USA.

[27] Hayes S., Velanis C.N., Jenkins G.I., Franklin K.A. (2014). UV-B detected by the UVR8 photoreceptor antagonizes auxin signaling and plant shade avoidance. Proc. Natl. Acad. Sci. USA.

[28] de Lucas M., Davière J.M., Rodríguez-Falcón M., Pontin M., Iglesias-Pedraz J.M., Lorrain S., Fankhauser C., Blázquez M.A., Titarenko E., Prat S. (2008). A molecular framework for light and gibberellin control of cell elongation. Nature.

[29] Feng S., Martinez C., Gusmaroli G., Wang Y., Zhou J., Wang F., Chen L., Yu L., Iglesias-Pedraz J.M., Kircher S. (2008). Coordinated regulation of Arabidopsis thaliana development by light and gibberellins. Nature.

[30] O’Hara A., Jenkins G.I. (2012). In vivo function of tryptophans in the Arabidopsis UV-B photoreceptor UVR8. Plant Cell.

[31] Nieto C., López-Salmerón V., Davière J.-M., Prat S. (2015). ELF3-PIF4 interaction regulates plant growth independently of the Evening Complex. Curr. Biol..

[32] Toledo-Ortiz G., Johansson H., Lee K.P., Bou-Torrent J., Stewart K., Steel G., Rodríguez-Concepción M., Halliday K.J. (2014). The HY5-PIF regulatory module coordinates light and temperature control of photosynthetic gene transcription. PLoS Genet..

[33] Ulm R., Baumann A., Oravecz A., Máté Z., Adám E., Oakeley E.J., Schäfer E., Nagy F. (2004). Genome-wide analysis of gene expression reveals function of the bZIP transcription factor HY5 in the UV-B response of *Arabidopsis*. Proc. Natl. Acad. Sci. USA.

[34] Brown B.A., Cloix C., Jiang G.H., Kaiserli E., Herzyk P., Kliebenstein D.J., Jenkins G.I. (2005). A UV-B-specific signaling component orchestrates plant UV protection. Proc. Natl. Acad. Sci. USA.

[35] Oravecz A., Baumann A., Máté Z., Brzezinska A., Molinier J., Oakeley E.J., Adám E., Schäfer E., Nagy F., Ulm R. (2006). CONSTITUTIVELY PHOTOMORPHOGENIC1 is required for the UV-B response in Arabidopsis. Plant Cell.

[36] Lee H.-J., Jung J.-H., Cortés Llorca L., Kim S.-G., Lee S., Baldwin I.T., Park C.-M. (2014). FCA mediates thermal adaptation of stem growth by attenuating auxin action in Arabidopsis. Nat. Commun..

[37] Galstyan A., Cifuentes-Esquivel N., Bou-Torrent J., Martinez-Garcia J.F. (2011). The shade avoidance syndrome in Arabidopsis: a fundamental role for atypical basic helix-loop-helix proteins as transcriptional cofactors. Plant J..

[38] Hornitschek P., Lorrain S., Zoete V., Michielin O., Fankhauser C. (2009). Inhibition of the shade avoidance response by formation of non-DNA binding bHLH heterodimers. EMBO J..

[39] Roig-Villanova I., Bou-Torrent J., Galstyan A., Carretero-Paulet L., Portolés S., Rodríguez-Concepción M., Martínez-García J.F. (2007). Interaction of shade avoidance and auxin responses: a role for two novel atypical bHLH proteins. EMBO J..

[40] Lorrain S., Trevisan M., Pradervand S., Fankhauser C. (2009). Phytochrome interacting factors 4 and 5 redundantly limit seedling de-etiolation in continuous far-red light. Plant J..

