# Supplementary material for: UV-B Perceived by the UVR8 Photoreceptor Inhibits Plant Thermomorphogenesis
Source: Curr Biol. 2017 Jan 9;27(1):120–7. doi: 10.1016/j.cub.2016.11.004 (PMC5226890; doi:10.1016/j.cub.2016.11.004)
Supplement: Document S1. Supplemental Experimental Procedures and Figures S1–S4 [file mmc1.pdf]

Current Biology, Volume *27*

Supplemental Information

UV-B Perceived by the UVR8 Photoreceptor Inhibits Plant Thermomorphogenesis

Scott Hayes, Ashutosh Sharma, Donald P. Fraser, Martine Trevisan, C. Kester Cragg-Barber, Eleni Tavridou, Christian Fankhauser, Gareth I. Jenkins, and Keara A. Franklin

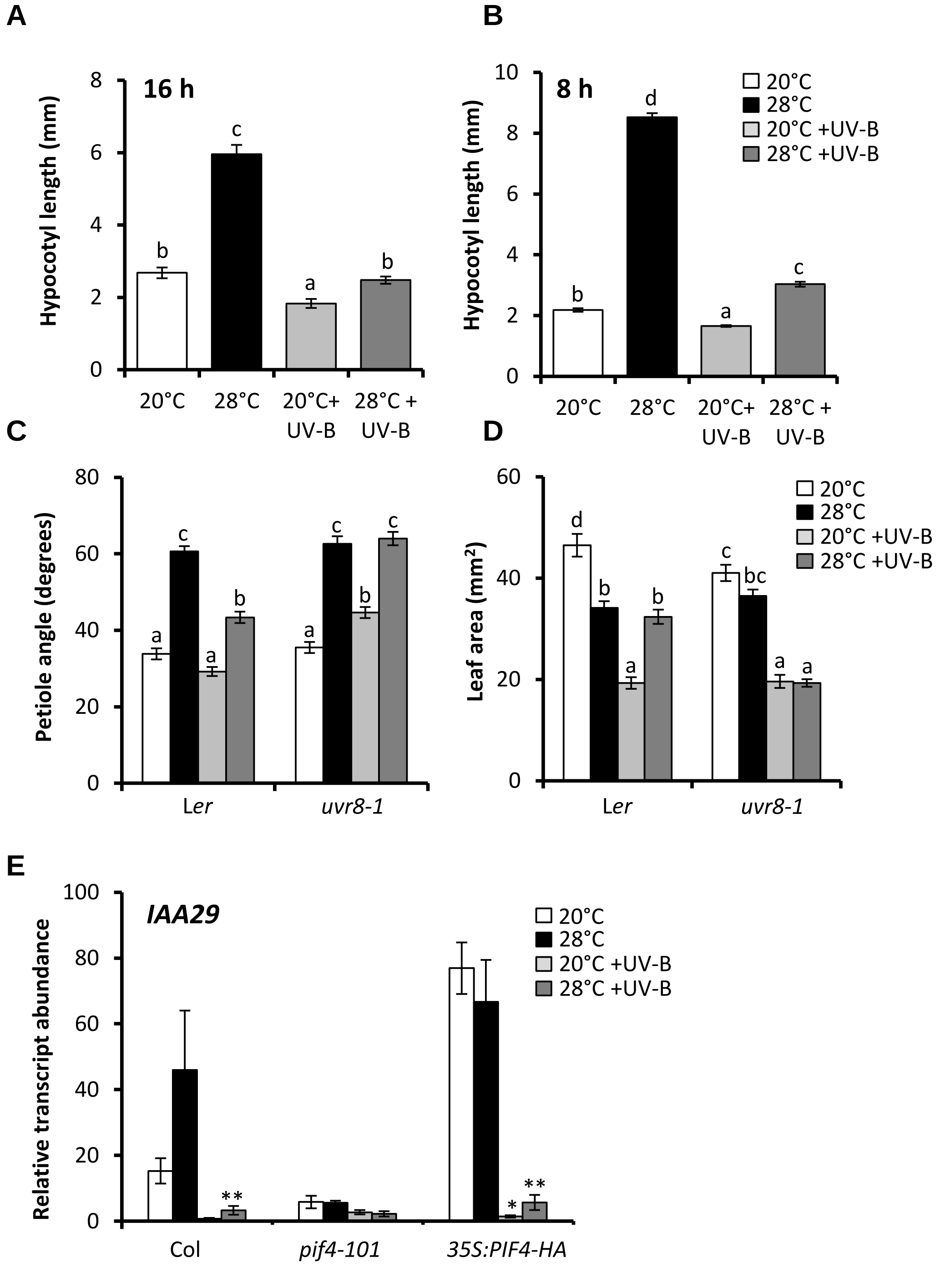

A
16 h
Hypocotyl length (mm)
8
6
4
2
0
b
c
a
b
20°C
28°C
20°C+ UV-B
28°C + UV-B
B
8 h
10
8
6
4
2
0
d
b
a
c
20°C
28°C
20°C +UV-B
28°C +UV-B
C
Petiole angle (degrees)
80
60
40
20
0
a
c
a
b
a
c
b
c
Ler
uvr8-1
D
Leaf area (mm²)
60
40
20
0
d
b
a
b
c
bc
a
a
Ler
uvr8-1
E
IAA29
Relative transcript abundance
100
80
60
40
20
0
**
*
**
Col
pif4-101
35S:PIF4-HA

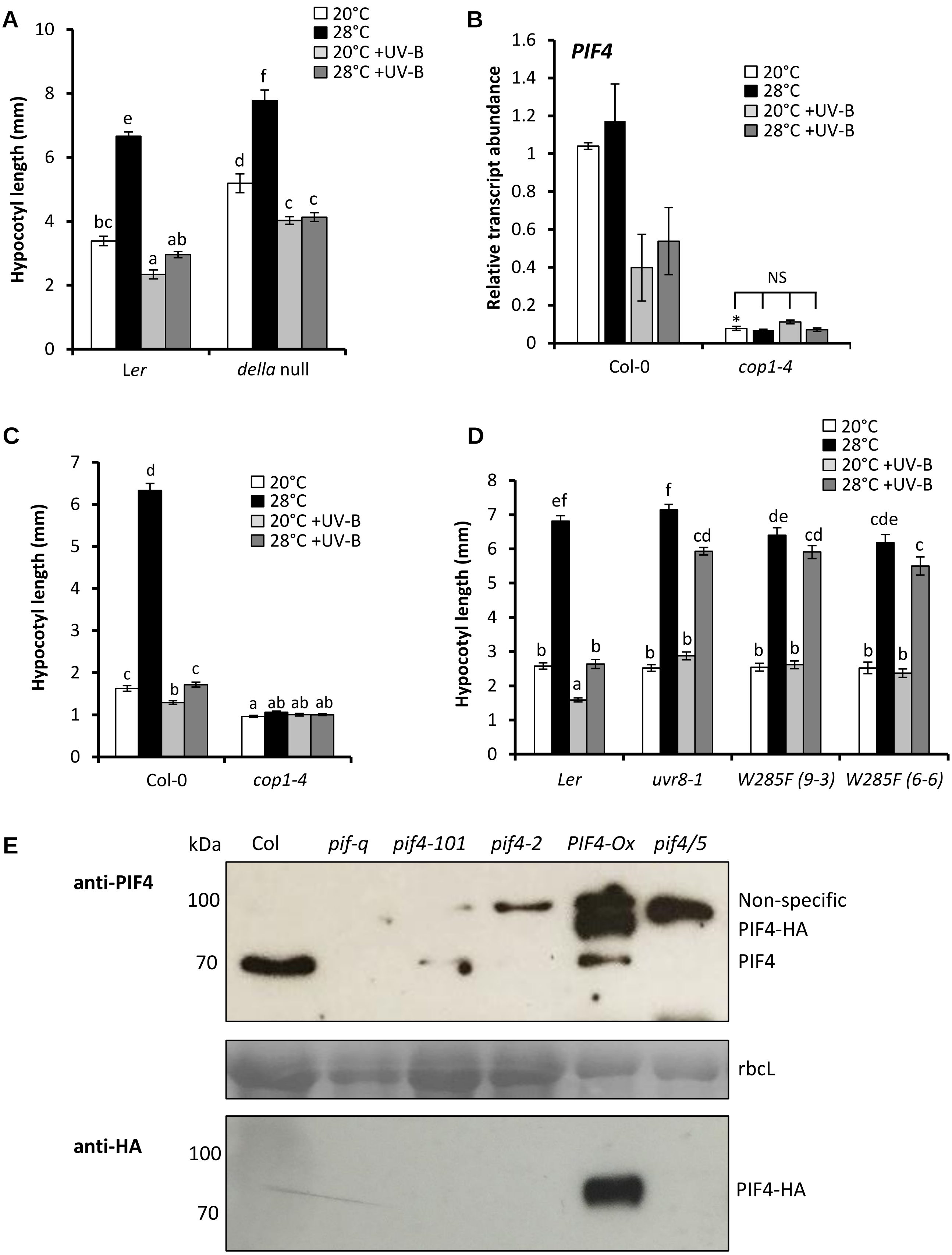
A
Hypocotyl length (mm)
20°C
28°C
20°C +UV-B
28°C +UV-B
bc
e
a
ab
d
f
c
c
Ler
della null
B
PIF4
Relative transcript abundance
20°C
28°C
20°C +UV-B
28°C +UV-B
NS
*
Col-0
cop1-4
C
Hypocotyl length (mm)
20°C
28°C
20°C +UV-B
28°C +UV-B
c
d
b
c
a
ab
ab
ab
Col-0
cop1-4
D
Hypocotyl length (mm)
20°C
28°C
20°C +UV-B
28°C +UV-B
b
ef
a
b
b
f
b
cd
b
de
b
cd
b
cde
b
c
Ler
uvr8-1
W285F (9-3)
W285F (6-6)
E
kDa
Col
pif-q
pif4-101
pif4-2
PIF4-Ox
pif4/5
anti-PIF4
100
70
Non-specific
PIF4-HA
PIF4
rbcL
anti-HA
100
70
PIF4-HA

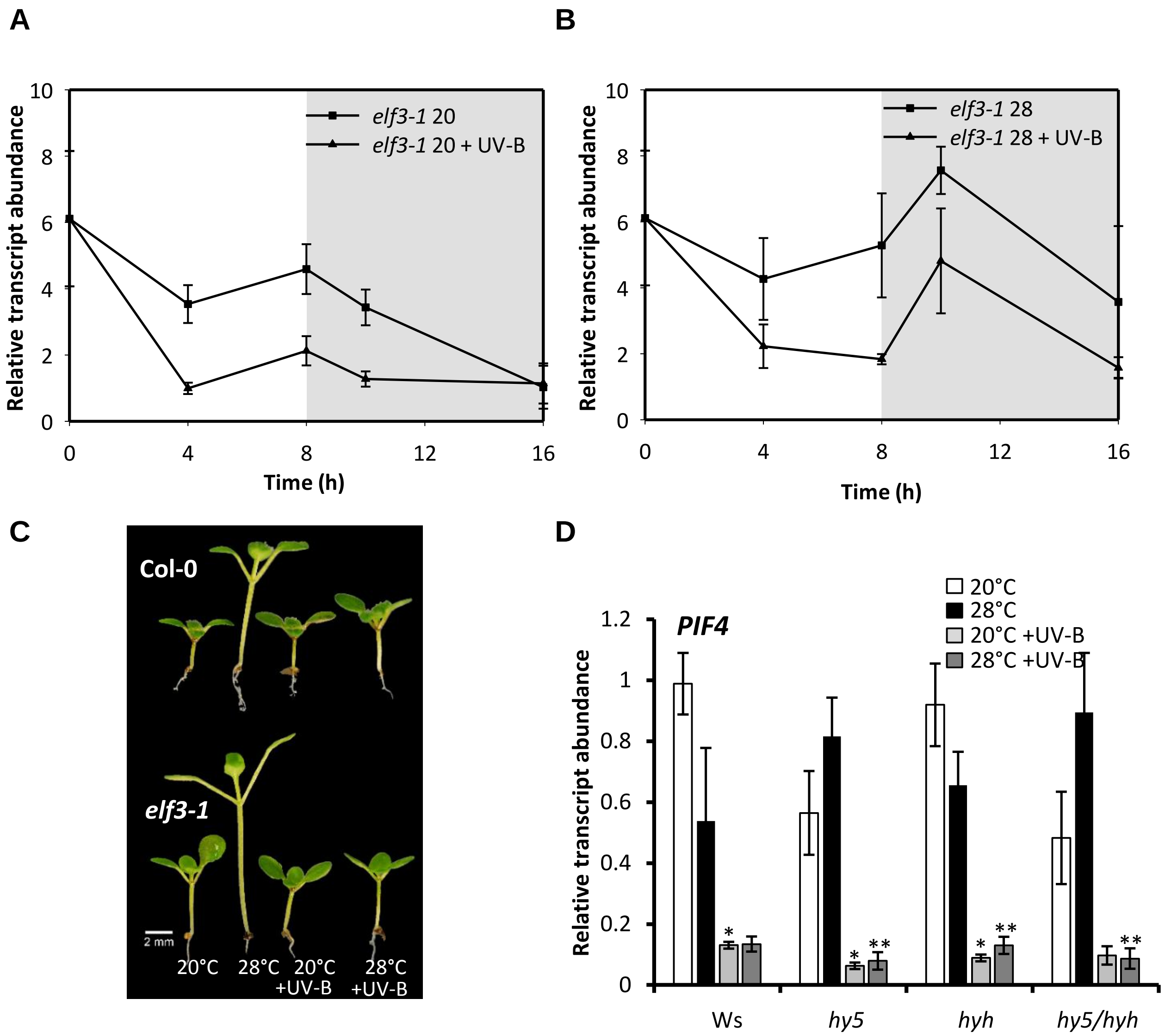
A
B
C
D
Relative transcript abundance
Time (h)
elf3-1 20
elf3-1 20 + UV-B
elf3-1 28
elf3-1 28 + UV-B
0
2
4
6
8
10
0
4
8
12
16
Col-0
elf3-1
2 mm
20°C
28°C
20°C +UV-B
28°C +UV-B
PIF4
20°C
28°C
20°C +UV-B
28°C +UV-B
0
0.2
0.4
0.6
0.8
1
1.2
Ws
hy5
hyh
hy5/hyh

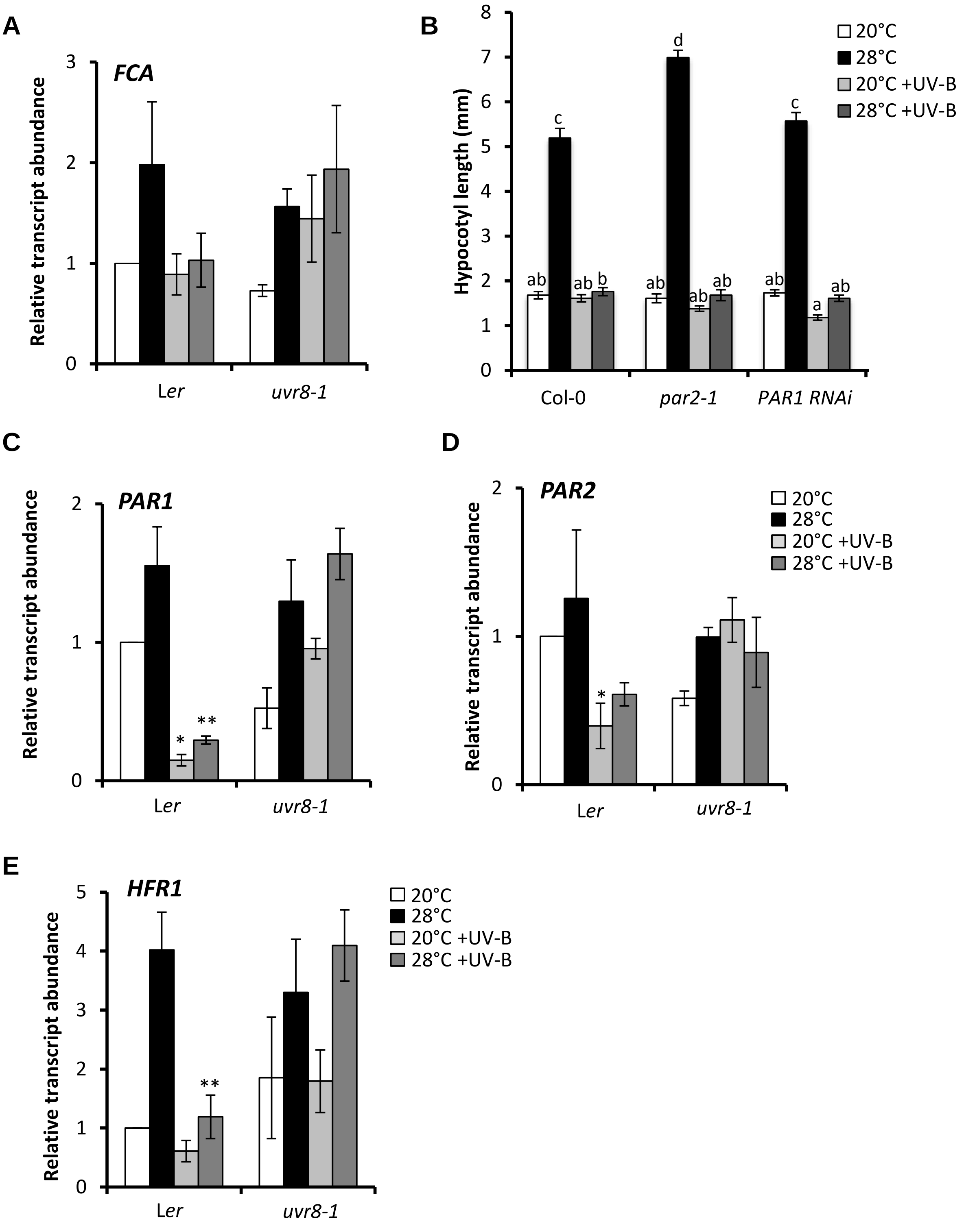
A
FCA
Relative transcript abundance
0
1
2
3
Ler
uvr8-1
B
Hypocotyl length (mm)
0
1
2
3
4
5
6
7
8
ab
c
ab
b
ab
d
ab
ab
ab
c
a
ab
Col-0
par2-1
PAR1 RNAi
20°C
28°C
20°C +UV-B
28°C +UV-B
C
PAR1
Relative transcript abundance
0
1
2
*
**
Ler
uvr8-1
D
PAR2
Relative transcript abundance
0
1
2
*
Ler
uvr8-1
20°C
28°C
20°C +UV-B
28°C +UV-B
E
HFR1
Relative transcript abundance
0
1
2
3
4
5
**
Ler
uvr8-1
20°C
28°C
20°C +UV-B
28°C +UV-B

Supplemental Figure Legends

Figure S1, related to Figures 1 and 2. UV-B inhibits plant photomorphogenesis in multiple photoperiods. (A,B) Hypocotyl lengths of Col-0 seedlings grown in (A) 16 h light/ 8 h dark cycles and (B) 8 h light/ 16 h dark cycles for 3 d at 20°C, before transfer to 20°C, 28°C, 20°C +UV-B or 28°C +UV-B for a further 4 d. Data represent means (n = 15) ± SE. Different letters indicate statistically significant means (p <0.05). (C) Petiole angle from horizontal of L*er* and *uvr8-1* plants grown in 16 h light/ 8 h dark cycles for 10 d at 20°C before transfer to 20°C, 28°C, 20°C +UV-B or 28°C +UV-B for a further 8 d. Data represent mean (n=24) ±SE. (D) Leaf area of leaf 4 of plants grown as in (C) 9 d after transfer. Data represent mean (n ≥ 23) ± SE. Different letters indicate statistically significant means (p <0.05). (E) Relative transcript abundance of *IAA29* in Col-0, *pif4-101* and *PIF4ox* seedlings grown for 10 d in a 16 h photoperiod at 20°C, before transfer at dawn to the indicated conditions for 4 h. n=3 ± SE. *Significant UV-B-mediated decrease in transcript abundance when compared to 20°C (p <0.05). **Significant UV-B-mediated decrease in transcript abundance when compared to 28°C (p <0.05).

Figure S2, related to Figures 3 and 4. UV-B-mediated suppression of high temperature-induced hypocotyl elongation is not mediated by DELLA proteins and COP1 promotes *PIF4* expression. (A) Hypocotyl lengths of L*er* and *della* null seedlings grown in continuous light for 3 d at 20°C, before transfer to 20°C, 28°C, 20°C +UV-B or 28°C +UV-B for a further 4 d. Data represent means (n ≥ 19) ±SE. Different letters indicate statistically significant means (p <0.05). 2-way ANOVA confirmed there was no significant interaction between genotype and condition on hypocotyl length (p >0.1). (B) Relative transcript abundance of *PIF4* in Col-0 and *cop1-4* seedlings grown for 10 days in 16 h light/ 8 h dark cycles at 20°C , before transfer at dawn to the indicated conditions for 4 h. n=3 ± S.E. *Significant decrease in transcript abundance when compared to Col-0 in identical conditions (p <0.05). NS = No significant difference in *PIF4* transcript abundance between indicated treatments (p <0.05). (C) Hypocotyl lengths of Col-0 and *cop1-4* seedlings grown in continuous light for 3 d at 20°C, before transfer to 20°C, 28°C, 20°C +UV-B or 28°C +UV-B for a further 4 d. n=35 ± SE. Different letters indicate statistically significant means (p <0.05). (D) Hypocotyl lengths of L*er*, *uvr8-1* and two independent lines of *uvr8-1*/GFP-UVR8^{W285F} (*W285F*), were grown in continuous light for 3 d at 20°C, before transfer to 20°C, 28°C, 20°C +UV-B or 28°C +UV-B for a further 4 d. n ≥ 10 ± SE. Different letters indicate statistically significant means (p <0.05). (E) PIF4 and PIF4-HA abundance in WT, PIF4-deficient mutants (*pif-q, pif4-101, pif4-2* and *pif4/5*) and *PIF4-HA* overexpressor plants grown for 10 d in 16 h light/ 8 h dark cycles at 20°C and harvested before dawn. The blot was probed with anti-PIF4, stripped and re-probed with anti-HA. Ponceau stain of Rubisco large subunit (rbcL) serves as a loading control.

Figure S3, related to Figure 3 and 4. UV-B-mediated suppression of high temperature-induced hypocotyl elongation is not mediated by ELF3 or HY5. (A,B) Time course of *PIF4* transcript abundance in *elf3-1* mutants. Seedlings were grown for 10 d in 8 h light/16 h dark cycles at 20ºC. On day 11, plants were transferred to either 20ºC or 28ºC ± UV-B. UV-B treatment was maintained for the duration of the photoperiod and plants harvested at the times shown. All values are normalized to Col-0 at time 0. The mean of 2 biological repeats are shown ± SE. (C) Col-0 and *elf3-1* seedlings grown in continuous light for 3 d at 20°C, before transfer to 20°C, 28°C, 20°C +UV-B or 28°C +UV-B for a further 4 d. (D) Relative transcript abundance of *PIF4* in Ws, *hy5*, *hyh* and *hy5/hyh* seedlings grown for 10 d in 16 h light/ 8 h dark cycles at 20°C, before transfer at dawn to the indicated conditions for 4 h. n=3 ± SE. *Significant UV-B-mediated decrease in transcript abundance when compared to 20°C (p <0.05). **Significant UV-B-mediated decrease in transcript abundance when compared to 28°C (p <0.05).

Figure S4, related to Figure 4. UV-B does not increase transcript abundance of *FCA*, *PAR1*, *PAR2* or *HFR1*. (A) Relative transcript abundance of *FCA* in L*er* and *uvr8-1* seedlings grown for 10 d in a 16 h light/ 8 h dark cycles at 20°C, before transfer at dawn to the indicated conditions for 4 h. n=3 ± SE. (B) Hypocotyl lengths of the *par2-1* mutant and *PAR1*-RNAi line grown in continuous light for 3 d at 20°C, before transfer to 20°C, 28°C, 20°C +UV-B or 28°C +UV-B for a further 4 d. Data represent means (n ≥ 19) ±SE. Different letters indicate statistically significant means (p <0.05). (C) Relative transcript of *PAR1*, (D) *PAR2* and (E) *HFR1* grown as in (A). *Significant UV-B-mediated decrease in transcript abundance when compared to 20°C (p <0.05). **Significant UV-B-mediated decrease in transcript abundance when compared to 28°C (p <0.05).

Supplemental Experimental Procedures

Plant material

All mutants and transgenic lines used in this study have been described previously. The *uvr8-1* [S1] and the *della* null [S2] mutants are in the Landsberg *erecta* (L*er*) background. The *hy5KS50* [S3], *hyh* [S4] and *hy5KS50/hyh* mutants [S4] are in the Wassilewskija (Ws) background. The *PIF4-HA* over-expressor, *pif4-101* and *pif4/5* lines [S5], *PAR1-RNAi* and *par2-1* lines [S6], *pif4-2* and *pif-q* mutants [S7], *elf3-1* [S8] and *cop1-4* [S9] mutants are in the Columbia (Col-0) background. *uvr8-1*/GFP-UVR8^{W285F} lines are in the L*er* background [S10]. The *hfr1-101* mutant is in the Col-7 background [S11]. pPH73 (*HFR1pro:HFR1-3XHA*) was constructed by amplifying 2.1kb 5' of the *HFR1* ATG using primers 5'-tgactctagaggtaccggcgatcgctacgaaaagaagaag-3' and 5'-gtcaggatccttagttaaagagatatcggagatga-3'. *HFR1* cDNA with a triple HA tag at the C-terminus was amplified from vector pCF396 described in [S12]. HFR1 promoter and cDNA were ligated into pPZP211 including an *RBCS* terminator sequence 3' of the *HFR1* gene. This construct was transformed into *hfr1-101* and lines with a single insertion site that complemented the *hfr1* phenotype were selected.

Growth conditions

Seeds were sown directly onto a 3:1 mixture of compost: horticultural silver sand. After 4 d stratification in darkness at 4ºC, seedlings were germinated in controlled growth cabinets (Microclima 1600E, Snijder Scientific) in continuous white light at 20˚C and 70% humidity. Plants were either left in control cabinets or transferred to the indicated conditions at the specified time. For adult plant experiments, qPCR analysis and western blots, plants were grown in the same cabinets under 16 h light/8 h dark cycles or 8 h light/ 16 h dark cycles. White light was provided by cool-white fluorescent tubes (400-700 nm) at a photon irradiance of 90 $\mu molm^{-2}s^{-1}$. Supplementary narrowband UV-B was provided at a photon irradiance of 400 mWm^2 (approximately 1 $\mu molm^{-2}s^{-1}$) by Philips TL100W/01 tubes. UV-B levels were modulated by strips of heat-proof tape. Biologically effective UV-B dose (BE-UV-B) was calculated 3.6 $\mu Wm^{-2}nm^{-1}$, following Flint and Caldwell [S13]. Control conditions also contained the same UV-B tubes, with an extruded acrylic cover which selectively blocked UV wavelengths. All light measurements were performed using an Ocean Optics FLAME-S-UV-VIS spectrometer with a cosine corrector (oceanoptics.com).

Plant measurements

Measurements of hypocotyl length, petiole length, leaf angle and leaf area were recorded using ImageJ software (http://www.rsb.info.gov/ij). For hypocotyl measurements, a minimum of 15 seedlings were measured for each genotype in each condition. For leaf area and petiole length measurements, the largest fully expanded rosette leaf (leaf 4) was excised from each plant at day 19. Leaf angles of leaf 4 were measured from the horizontal soil surface at day 18. Measurements were recorded from a minimum of 23 plants per treatment. At least two biological repeats were performed for each experiment with similar results.

RNA extraction and qPCR analysis.

Seedlings were initially grown in control conditions under 16 h light/ 8 h dark cycles for 10 d, before transfer at dawn to different light and temperature conditions for the indicated time. RNA extraction, cDNA synthesis and qPCR were performed as described previously [S14]. Transcript abundance values were normalised to *ACTIN2*. See list of primers for *ACTIN2*, *FCA*, *HFR1*, *IAA29*¸ *PAR1*, *PAR2*, *PIF4*, and *YUCCA8* sequences. Three biological replicates were performed for each experiment. For time course analyses, 2 biological repeats were performed with similar results.

Protein extraction and western blotting

Seedlings were grown in 16 h light/ 8 h dark photoperiods for 10 d at 20°C, before transfer at dawn to different light and temperature conditions for the indicated time. Samples were harvested into liquid nitrogen and extracted in buffer (100 mM tris-HCl (pH 8), 4 M urea, 5% (w/v) SDS, 15% (v/v) glycerol, 10 mM β-ME, 30 µl/ml protease inhibitor cocktail (Sigma)), before boiling at 95°C for 4 min and centrifugation at maximum speed for 15 min. Protein levels were quantified by RC DC Lowry assay (Biorad). SDS-PAGE sample buffer (4x (8% (w/v) SDS, 0.4% (w/v) bromophenol blue, 40% (v/v) glycerol, 200 mM tris-HCl (pH6.8), 400 mM β-ME)) was added to supernatants to a final dilution of 1x. Samples were then heated for 5 min at 95°C.

For PIF4-HA immunoblots, 30 µg of total protein was loaded on to 10% SDS-PAGE gels and blotted on to nitrocellulose membranes (Biorad). Membranes were incubated overnight at 4°C in 1:1000 anti-HA antibody conjugated to peroxidase (Roche). Signals were detected using ECL2 (Thermo Scientific). Blots were performed in triplicate at each time point. Band intensity was analysed with ImageJ. Protein abundance was normalised to ponceau staining of rubisco large subunit and expressed as a value relative to pre-dawn levels.

For native PIF4 and HFR1 immunoblots, protein extracts (70 µg for PIF4 or 40 µg for HFR1-HA) were separated on 10% or 12% SDS-PAGE gels, respectively and blotted on PVDF membrane (Thermo Scientific). Membranes were incubated overnight at 4ºC in 1:1000 anti-PIF4 (Agrisera) or 1:2000 anti-HA (Covance). Secondary antibody incubations were performed for 1 h at room temperature using 1:10000 anti-rabbit (Promega) or 1:5000 anti-mouse (Dako) antibodies conjugated with peroxidase. Signals were detected using the SuperSignal™ West Femto Maximum Sensitivity Substrate (Thermo Scientific). Protein abundance was normalised to ponceau staining of rubisco large subunit.

Statistical analyses

Statistical analyses were carried out using IBM SPSS Statistics 21.0 software. Morphological assays were analyzed using a one-way ANOVA, treating genotype and temperature/light condition together as a single factor. Tukey's post-hoc tests were used to deduce statistically significant means ($p < 0.05$) as indicated by letters in the figures. For selected experiments 2-way ANOVAs were performed to either confirm or rule-out interactions between genotypes and conditions. For qPCR analyses, relative expression values were first transformed by Log2. Student's t-tests were then performed to investigate differences between the means indicated in the figure legends ($p < 0.05$). Student's t-test was also performed to analyse quantitative western blot data.

Primer sequences used for qPCR

**Primer**	**Sequence**
ActinF	TCAGATGCCCAGAAGTGTTGTTCC
ActinR	CCGTACAGATCCTTCCTGATATCC
FCAF	GCTCTTGTCGCAGCAAACTC
FCAR	GATCCAGCCCACTGTTGTTTAC
HFR1F	TAAATTGGCCATTACCACCGTTTA
HFR1R	ACCGTGAAGAGACTGAGGAGAAGA
IAA29F	ATCACCATCATTGCCCGTAT
IAA29R	ATTGCCACACCATCCATCTT
PAR1F	CACGAGACGCTCTCTGT
PAR1R	TTCTCGGTCTTCACGTAC
PAR2F	CGTAGAAGATGAAGATGAA
PAR2R	CGTAGTAAGAACTTTAATGG
PIF4F	GCCGATGGAGATGTTGAGAT
PIF4R	CCAACCTAGTGGTCCAAACG
YUCCA8F	ATCAACCCTAAGTTCAACGAGTG
YUCCA8R	CTCCCGTAGCCACCACAAG

Supplemental References

S1. Kliebenstein, D., Lim, J., Landry, L., and Last, R. (2002) Arabidopsis UVR8 regulates Ultraviolet-B signal transduction and tolerance and contains sequence similarity to Human Regulator of Chromatin Condensation 1. Plant Physiol. *130*, 234–43.

S2. Koini, M.A., Alvey, L., Allen, T., Tilley, C.A., Harberd, N.P., Whitelam, G.C., and Franklin, K.A. (2009). High Temperature-Medated Adaptations in Plant Architecture Require the bHLH Transcription Factor PIF4. Curr. Biol. *19*, 408–413.

S3. Oyama, T., Shimura, Y., and Okada, K. (1997) The Arabidopsis HY5 gene encodes a bZIP protein that regulates stimulus-induced development of root and hypocotyl. Genes Dev. *11*, 2983–95.

S4. Holm, M., Ma, L., Qu, L., and Deng, X-W (2002) Two interacting bZIP proteins are direct targets of COP1-mediated control of light-dependent gene expression in Arabidopsis. Genes Dev. *16*, 1247–59.

S5. Lorrain, S., Allen, T., Duek, P., Whitelam, G.C, and Fankhauser C. (2008) Phytochrome-mediated inhibition of shade avoidance involves degradation of growth-promoting bHLH transcription factors. Plant J. *53*, 312–23.

S6. Roig-Villanova, I., Bou-Torrent, J., Galstyan, A., Carretero-Paulet, L., Portolés, S., Rodríguez-Concepción, M. and Martínez-García, J.F. (2007). Interaction of shade avoidance and auxin responses: a role for two novel atypical bHLH proteins. EMBO J. *26*, 4756-4767.

S7. Leivar, P., Monte, E., Oka, Y., Liu, T., Carle, C., Castillon, A., Huq, E. and Quail, P.Q. (2008). Multiple phytochrome-interacting bHLH transcription factors repress premature seedling photomorphogenesis in darkness. Curr. Biol. *18*, 1815–1823.

S8. Liu, X., Covington, M., Fankhauser, C., Chory, J. and Wagner, D. (2001) ELF3 encodes a circadian clock-regulated nuclear protein that functions in an Arabidopsis PHYB signal transduction pathway. Plant Cell *13*, 1293–304.

S9. McNellis, T., von Arnim, A., Araki, T., Komeda, Y., Miséra, S., and Deng, X. (1994) Genetic and molecular analysis of an allelic series of cop1 mutants suggests functional roles for the multiple protein domains. Plant Cell *6*, 487–500.

S10. O'Hara, A., and Jenkins, G.I. (2013) In vivo function of tryptophans in the Arabidopsis UV-B photoreceptor UVR8. Plant Cell *24*, 3755-3766.

S11. Duek, P. and Fankhauser, C. (2003) HFR1, a putative bHLH transcription factor, mediates both phytochrome A and cryptochrome signalling. Plant J. *34*, 827–36.

S12. Duek P.D., Elmer, M.V., van Oosten, V.R., Fankhauser, C. (2004) The Degradation of HFR1, a Putative bHLH Class Transcription Factor Involved in Light Signaling, Is Regulated by Phosphorylation and Requires COP1. Current Biology *14*, 2296–2301.

S13. Flint, S., and Caldwell, M. (2003) A biological spectral weighting function for ozone depletion research with higher plants. Physiol. Plant. *117*, 137–44.

S14. Salter M.G., Franklin, K.A., Whitelam, G.C. (2003) Gating of the rapid shade-avoidance response by the circadian clock in plants. Nature *426*, 1–4.
